# The lexical profile of forestry academic texts: What does it take to understand a specialized discipline?

**DOI:** 10.1371/journal.pone.0315975

**Published:** 2024-12-30

**Authors:** Guihua Luo, Jiamiao Song, Jingjing Wu

**Affiliations:** 1 College of Foreign Languages, Central South University of Forestry and Technology, Changsha, Hunan, China; 2 Institute of Language Sciences, Shanghai International Studies University, Shanghai, China; 3 College of Foreign Studies, Guilin University of Technology, Guilin, Guangxi, China; 4 Institute for Regional Economic and Language Service Research, Guilin, Guangxi, China; The University of Auckland, NEW ZEALAND

## Abstract

Vocabulary is essential for comprehension, especially in specialized disciplines. However, the research on the lexical features of forestry remains underexplored. This study focuses on the vocabulary frequency profile of forestry academic texts, and compares forestry vocabulary with general vocabulary and general academic vocabulary. Using Range software and the BNC/COCA word lists, this research analyzed a corpus of 331 research articles and 14 textbooks across eight forestry sub-disciplines. Results demonstrate a rich and diverse vocabulary in this discipline. Two forestry-specific word lists, the forestry Latin loan word list and the forestry English word list, were developed from words not included in the BNC/COCA word lists. The analysis indicates that mastering the top 5,000 word families and four supplementary word lists from the expanded BNC/COCA word lists provides 95% lexical coverage of the forestry academic corpus. For 98% coverage, prioritizing the two forestry-specific word lists reduces the required word families from 11,000 to 8,000. While both forestry research articles and textbooks need 5,000 word families for 95% coverage, research articles require an additional 2,000 for 98% coverage. Lexical demands across sub-disciplines range from 4,000 to 6,000 word families for 95% coverage, and from 8,000 to 11,000 for 98% when including the forestry-specific word lists. Furthermore, findings also indicate that the forestry vocabulary contains a higher proportion of mid- and low-frequency words than general vocabulary and general academic vocabulary. These findings provide important guidance for setting vocabulary learning goals and sequences for learners in the forestry discipline, thereby contributing to improved comprehension of forestry academic texts and enhancing the effectiveness of English for Specific Purposes instruction.

## 1. Introduction

Vocabulary is the cornerstone of language and is crucial for both expression and comprehension [1]. Rich vocabulary knowledge contributes to enhancing text comprehension, whereas a limited vocabulary can pose significant difficulties [2]. Given the vast vocabulary in the English language, vocabulary learning becomes one of the biggest challenges that students face in their reading comprehension. Not surprisingly, then, teachers and learners are concerned with identifying the essential vocabulary for effective reading comprehension and determining which words are pertinent for specific purposes. Studies on word lists provide valuable insights into addressing this issue. For example, novice learners across different age groups are recommended to focus on mastering the initial 2,000 words listed in the General Service List (GSL) [3]. For middle school learners and beyond who have achieved proficiency in much of the GSL, the Academic Word List (AWL) [4] becomes a critical objective and resource for vocabulary learning [5]. The 2,000 word families in the GSL and the 570 word families in the AWL collectively account for nearly 90% of the running words in most academic texts [6].

After mastering the vocabulary in the GSL and the AWL, learners, particularly those in college or university settings, are recommended to focus on acquiring vocabulary tailored to their specific subject areas [5,7]. Meanwhile, it has been found that English for Specific Purposes (ESP) learners encounter substantial challenges in acquiring and mastering specialized vocabulary in their academic or professional domains [8,9]. In recent decades, researchers in vocabulary studies have developed some discipline-specific word lists to help with vocabulary learning and understanding in specialized disciplines [10–17]. Studies have also documented lexical profiles of various disciplines, including business [7], engineering [10], traditional Chinese medicine [13], physics [18], and marine engineering [19]. This body of work has identified the necessary quantity and specific vocabulary essential for achieving proficiency across several disciplines, and has highlighted the differing lexical demands [20].

However, the need for more word lists and vocabulary profile analyses across additional disciplines remains critical to provide principled, discipline-specific vocabulary knowledge for ESP learners. Forestry is a fundamental academic discipline characterized by its multidisciplinary nature that integrates ecology, management, and conservation to promote sustainable forest resource practices and address environmental challenges [21,22]. Given the scarcity of research on vocabulary in forestry, this study investigates the vocabulary used in forestry academic contexts. The goal is to provide a comprehensive understanding of the vocabulary needed for reading comprehension in this specialized discipline. To achieve this purpose, the lexical profile of forestry academic vocabulary will be analyzed both independently and in comparison, with general vocabulary and general academic vocabulary.

## 2. Literature review

### 2.1 Lexical coverage, lexical demands and lexical profile

Lexical coverage is defined as “the percentage of running words in the text known by readers” [23]. Studies in lexical coverage have explored the relationship between vocabulary knowledge and reading comprehension to find the minimal vocabulary required for “adequate” and “comfortable” reading comprehension. The lexical coverage thresholds frequently cited are 95% for adequate comprehension and 98% for comfortable comprehension [24–27].

Lexical demands refer to the number of words that learners must understand to meet the threshold required for adequate (95%) or comfortable (98%) comprehension [28]. Therefore, the issue of lexical demands is addressed by asking how many words in a text or corpus need to be understood for adequate or comfortable comprehension [23,29–31]. Accordingly, different types of texts require readers to know varying amounts of vocabulary to adequately or comfortably understand them, and larger lexical knowledge would be needed to comprehend more challenging texts [23,32].

Lexical profile provides an indication of the difficulty level of a text, the potential for vocabulary learning through reading or listening to the text, as well as the vocabulary knowledge required to understand the text, or different types of texts [33]. Technological innovations in computer software and advancements in corpus linguistics have greatly facilitated this type of vocabulary analysis. Researchers can conduct corpus-driven analysis of multi-million-word corpora, which enables them to create comprehensive word frequency lists. These lists, combined with specialized software, allow for the profiling of word distribution across different frequency levels within various discourses [34]. Lexical profile analysis, which is based on word lists and employs a corpus-driven approach [13,30,31,34], provides insights into the coverage of high-, mid-, and low-frequency words within specialized discourses. These studies are particularly beneficial for establishing vocabulary learning targets, which indicate when a discipline can be understood. Lexical profile studies also highlight the relative importance of specific words in different types of discourse, assess the potential for vocabulary acquisition through reading or listening to the discourse, and identify the vocabulary knowledge required to comprehend various forms of spoken and written discourse [35].

### 2.2 Lexical coverages for reading comprehension

Vocabulary often serves as a reliable predictor of reading comprehension [25,36]. Several studies on lexical coverage have investigated the correlation between vocabulary knowledge and reading comprehension and determined the necessary vocabulary proportion for adequate and comfortable comprehension. Liu and Nation discovered that knowing 95% of the vocabulary in a given text or corpus is essential for accurately inferring words in context [37]. Similarly, Laufer found that learners need to understand a minimum of 95% of the vocabulary in written texts to achieve sufficient comprehension. Some studies have suggested a higher threshold [24,38]. Hirsh and Nation revealed that 98% coverage is needed for learners to read for pleasure [39]. Hu and Nation also reported that when learners are acquainted with 95% of the running words in a text, they will encounter an unfamiliar token approximately every 20 words [25]. However, this ratio will diminish to 1/50 if they are familiar with 98% of the tokens, which means a 98% coverage may be “the ideal coverage” because for the residual percentages of unfamiliar words within a text, readers can employ contextual cues for inference or resort to a dictionary without external aid [23]. Thus, it is widely acknowledged that 95% coverage is considered the minimum for reading comprehension, while 98% represents the optimal level. These established thresholds have sparked discussions in English for academic purposes (EAP) teaching, as they allow for the calculation of the vocabulary required to comprehend specialized discipline texts [40].

### 2.3 Lexical demands of discipline-specific texts

Studies of lexical profiles have garnered interests and provided vocabulary learning targets for certain disciplines or registers. Table 1 shows the lexical demands of the specialized disciplines studied.

**Table 1:** Past studies of lexical demands of discipline-specific texts.

Previous research	Text types	Lexical demands at 95% coverage	Lexical demands at 98% coverage
[7]	Business textbooks	3,500 + PN	5,000 + PN
	Business research articles	5,000 + PN	8,000 + PN
[10]	Engineering textbooks	5,000 + PN, TC, AC	10,000+ PN, TC, AC
[18]	Physics textbooks	8,000 + PN, MW, AC	NA
	Physics research articles	NA	NA
	Physics lectures	4,000 + PN, MW, AC	10,000 + PN, MW, AC
	Physics magazines	NA	NA
	Physics popular books	4,000 + PN, MW, AC	10,000 + PN, MW, AC
	Physics TV documentaries	4,000 + PN, MW, AC	8,000 + PN, MW, AC
	Physics TED talks	4,000 + PN, MW, AC	7,000 + PN, MW, AC
[13]	Traditional Chinese medicine theory-based textbooks	7,000+ PN, MW, TC, AC + TCM base word lists	13,000+ PN, MW, TC, AC + TCM base word lists
	Traditional Chinese medicine practice-based textbooks	8,000+PN, MW, TC, AC + TCM base word lists	13,000+ PN, MW, TC, AC + TCM base word lists
	Traditional Chinese medicine research articles	7,000+ PN, MW, TC, AC + TCM base word lists	12,000+PN, MW, TC, AC + TCM base word lists
[9]	Marine engineering instruction books and manuals	12,000+ PN, MW, AC	NA

These studies have clarified that lexical demands vary significantly across academic disciplines and even differ within specific genres of each discipline. As shown in Table 1, when aiming for 95% coverage, Business textbooks require a minimum of 3,500 word families plus proper nouns (PN), while business research articles necessitate at least 5,000 word families plus proper nouns [10]. Traditional Chinese medicine (TCM) presents particularly high lexical demands, as the British National Corpus/ Corpus of Contemporary American English (BNC/COCA) word lists have been found insufficient to meet the 95% threshold. This necessitates the use of the two self-developed TCM base word lists [13]. When aiming for 98% coverage, business exhibits the lowest lexical demands, requiring 5,000 and 8,000 word families plus proper nouns for textbooks and research articles, respectively. In marine engineering instruction books and manuals, the BNC/COCA word lists have been found insufficient to meet the 98% threshold [9], denoted by “NA” in Table 1. The BNC/COCA word lists alone are also insufficient to achieve 98% coverage in traditional Chinese medicine. Research articles require 12,000 word families plus four supplementary word lists, including proper nouns, marginal words (MW), transparent compounds (TC), acronyms (AC), and two TCM base word lists. For theory-based and practice-based textbooks, 13,000 word families plus four supplementary word lists are necessary, along with the same TCM base word lists, to attain 98% coverage. In other words, traditional Chinese medicine research articles impose slightly lower lexical demands compared to textbooks. However, the other disciplines have a different picture as research articles have higher lexical demands than textbooks.

Previous research has demonstrated how to make use of the existing word lists, especially the BNC/COCA word lists, to understand the vocabulary and lexical demands of specific genres or disciplines. Lexical profile analysis effectively describes and illustrates the vocabulary characteristics and text difficulty of a particular discipline, thereby determining the target of vocabulary knowledge required for students to comprehend the texts. Additionally, it enables the comparison of the words used in different disciplines and genres. While various studies have been conducted to investigate the lexical profile of specialized disciplines, the lexical profile of forestry academic texts remains underexplored. However, forestry plays a crucial role in sustainable resource management, biodiversity conservation, and addressing climate change [21,22]. It is essential to understand the specialized vocabulary used in this field.

### 2.4 The BNC/COCA word lists

Lexical profile research is based on word frequency lists, including general high-frequency word lists, such as the GSL [3], and general academic word lists, such as the AWL [4]. These lists classify English words into several 1,000-word levels according to how frequently they appear in authentic texts, which offers teachers and learners a clear and effective route to their learning goal [1]. In the lexical profile research of specialized disciplines, as illustrated in Table 1, the most widely used word list is the BNC/COCA word lists. The lists have served as a valuable resource for vocabulary acquisition since their development, and are regarded as the most efficient pathway to achieve the pedagogical or learning objectives [41–44].

The BNC/COCA word lists, created by Nation, were systematically and continuously developed according to frequency and range from a corpus totaling 10 million words—6 million spoken and 4 million written—sourced from the British National Corpus (BNC), the Corpus of Contemporary American English (COCA), and the Wellington Corpus of Spoken New Zealand English (WSC) [34]. These lists represent Nation’s refinement and improvement of the limitations in BNC2000 [23]. The unit of counting in the BNC/COCA word lists is the word-family, which includes a root word along with its inflectional and transparent derivational forms [1]. The BNC/COCA word lists comprise 25 base word lists, from the most frequent 1,000 word families to the low frequency 25,000 word families, and four supplementary lists of proper nouns (basewrd31), marginal words (basewrd32), transparent compounds (basewrd33), and acronyms (basewrd34). The BNC/COCA word lists enable researchers to examine vocabulary from a broad perspective. By analyzing how many words in the target corpus exist at various frequency levels and how many lexical items fall outside the frequency-based lists, we can assess the vocabulary difficulty of the corpus and, further, the lexical demands of the discipline.

## 3. Methodology

### 3.1 Research questions

Forestry, an important academic field, plays a critical role in addressing global challenges related to environmental sustainability, biodiversity conservation, and climate change mitigation [21,22]. This field has attracted universities worldwide to offer related courses and research opportunities aimed at cultivating professionals capable of addressing the challenges in forest resource management and conservation. Globally, hundreds of universities offer programs or majors related to forestry. Specifically, in China, approximately 50 universities offer forestry-related majors or degree courses. Despite forestry’s significance as a fundamental academic discipline, there is a lack of research into its lexicon. Motivated by the vocabulary needs of learners in this discipline, this study conducts a lexical profile analysis of forestry. The aims are to explore what kind of words make up the vocabulary in this specialized discipline, determine the coverage of the BNC/COCA word lists in the forestry academic corpus (FAC), and identify the vocabulary size necessary to reach 95% and 98% coverage of forestry academic English. Furthermore, the study seeks to compare forestry vocabulary with general vocabulary and general academic vocabulary.

The present research employs the BNC/COCA word lists and Range to investigate and analyze the vocabulary used in forestry academic journals and textbooks. The study addresses the following three research questions:

1. What is the lexical profile of the forestry academic corpus based on the BNC/COCA word lists?
2. What are the lexical demands for learners reading forestry academic texts?
3. To what extent does forestry vocabulary differ from general vocabulary and general academic vocabulary?

### 3.2 Data

To provide a vocabulary profile of the discipline of forestry, a forestry academic corpus was developed. The forestry academic corpus comprises two roughly equal parts: journal articles and textbooks. The journal articles were sourced from the Forest Science Database, a premier bibliographic database for research in forest science, wood science, and agroforestry worldwide. This database serves as a centralized platform for storing, accessing, and managing a vast array of scholarly resources related to forestry. We followed the Forest Science Database’s categorization of forestry into eight sub-disciplines: agroforestry, arboriculture, dendrochronology, forest economics, forest environment, forest products, forest trees, and forest management. On the one hand, journal articles from these eight sub-disciplines were retrieved through subject categorization techniques in the database to ensured that the criterion of topical relevance was met. The topic of the articles matches the corresponding sub-disciplines. To ensure recency, only articles published between 2018 and 2023 were included, and the selection was further refined by limiting the articles to SCI-indexed articles, satisfying the criteria of impact and usage criteria. On the other hand, the textbooks were globally sourced through researchers’ institutions, with the selection criteria including topical relevance, institutional influence (published by a reputable publisher), and current usage (with preference given to textbooks with higher usage counts in surveyed universities). Following selection, the mean token count for all collected texts within each sub-discipline was calculated, and texts with token counts greatly deviating from this mean count were excluded from the final selection. In total, the FAC comprised 331 research articles and 14 textbooks.

After selecting the specific texts for inclusion in the FAC and each sub-discipline, we processed the final data. This process began with the removal of the non-essential elements, such as author names, institutional affiliations, figures, annotations, URLs, headers, footers, page numbers, funding information, acknowledgments, and references in the research articles. Similarly, in textbooks, we removed cover details, author introductions, prefaces, figures, annotations, and references. After using Adobe Acrobat to eliminate these elements, we converted the original PDF format files of the journal articles and textbooks into.docx format, and MS Word was then used for thorough cross-checking to ensure the consistency and completeness of the removal and conversion process. Subsequently, the.docx files were converted to text files of.txt format for further analysis. Finally, all texts were classified into eight sub-disciplines, resulting in a total of 16 sub-corpora (eight categories of journal articles and eight categories of textbooks). These 16 sub-corpora are roughly evenly sized, and the information about those eight sub-disciplines is provided in Table 2.

**Table 2:** The composition of the forestry academic corpus.

Sub-corpus	Research articles			Textbooks	
	Number of texts	Number of tokens		Number of texts	Number of tokens
Agroforestry	44	219,668		2	220,686
Arboriculture	40	215,321		1	239,146
Dendrochronology	45	211,434		3	199,366
Forest Economics	40	217,270		1	210,527
Forest Environment	39	214,334		2	192,648
Forest Products	47	225,819		2	262,542
Forest Trees	39	243,536		2	199,148
Forest Management	37	216,064		1	198,177
**Total**	**331**	**1,763,446**		**14**	**1,722,240**

### 3.3 Instrument

The computer software Range, created by Nation and Heatley [34], and the BNC/COCA word lists were used in this study. The BNC/COCA word lists were originally made to be used with Range, so this study employed Range as the software. Range, crafted and developed by Paul Nation, is a powerful corpus analysis tool capable of extracting frequency and range data from a number of large corpora simultaneously. It is pre-loaded with the GSL and the AWL, and allows for the incorporation of additional word lists, such as the BNC/COCA word lists. The vocabulary level of a corpus can be extrapolated by comparing the words in the target corpus with those of the ranked BNC/COCA word family lists. In this study, Range was used to analyze the coverage and distribution of the BNC/COCA word lists in FAC and its eight sub-disciplines, and to compare and present vocabulary coverage in the FAC and the British Academic Written English (BAWE) corpus. The BAWE corpus is a large, formally compiled collection of academic writing samples from British university students. It contains over 6.5 million words from 6,000 texts across four major disciplines: arts and humanities, social sciences, life sciences, and physical sciences. Widely used as a resource for linguistic research, language teaching, and the development of writing skills, the corpus offers valuable insights into authentic academic language use [45].

### 3.4 Manual adaptation

Nation alerted researchers that the BNC/COCA word lists would always remain a piece of unfinished work, and the expansion of the BNC/COCA word lists is ongoing [34]. In this study, we found that the adaptation and expansion of the existing BNC/COCA word lists were necessary. After running FAC over the Range software, we identified 38,975 word types not yet included in the existing BNC/COCA word lists. The words put into the category of “not in the lists” by Range included proper nouns (e.g., *Coorg*), marginal terms (e.g., *P*_*max*_), acronyms (e.g., *TLS*), and a considerable number of hyphenated words (e.g., *community-based*). Additionally, many words closely related to existing word families were not included by the BNC/COCA word lists. These findings underscore the need to adapt the existing BNC/COCA word lists and reclassify the remining word types to ensure that the “not in the list” word types are categorized into the appropriate lists. To address this, the manual adaptation was conducted collaboratively by the research team, which includes two professors specializing in forestry. We crossed-checked the “not in the list” vocabulary by searching the corpora and consulting dictionaries (e.g. the Oxford Advanced Learner’s Dictionary), to verify: (1) whether they were errors generated during text conversion or processing; (2) whether they belonged to existing word families in BNC/COCA word lists; (3) whether they were foreign words borrowed from other languages; (4) whether they were specialized words closely related to the discipline. The adapting and categorizing of the remaining “not in the list” word types proceeded as follows:

Firstly, following Nation’s definition of word families [34], we added words not in the BNC/COCA word lists but that share the same base or root and have related meanings to the relevant existing word families in the 1–25 base word lists. For example, the word *unfired* was not included in the original lists and was added to the word family *fire*. All the proper nouns, marginal words, and acronyms in the corpus were categorized into basewrd31, basewrd32, and basewrd34, respectively. Through this process, the existing BNC/COCA word lists were adapted, and a large number of the “not in the list” word types were categorized according to the word lists to which they belonged.

Secondly, there was a considerable number of hyphenated words in the corpus that were listed in the category of “not in the list” by Range. In previous study [28], the hyphens in hyphenated words were simply replaced with space, which would have negative effects on vocabulary classification and word family frequency calculations. For example, the compound word *acid-base* also appeared as *acidbase* in corpus, so replacing hyphen with space would have counted *acid-base* as two different lexical items. To avoid this, we manually double-checked all the hyphenated words. Words (e.g., *agro-forestry*) related to the existing word family in 1–25 base word lists were added to the BNC/COCA word lists, with hyphens removed. We then inspected the remaining words to determine if they are transparent compounds i.e., words whose meaning is closely and clearly related to the meanings of their components (e.g., *acid-base*). If so, the hyphen was removed, and they were placed in basewrd33, the transparent compounds list. If not, the hyphen was removed, and the words (e.g. *farmer-perceptions*) were reclassified as corresponding separate lexical items. This manual adaptation offered dual benefits. For one thing, it added words to the 1–25 base word lists or the transparent compounds list. For another, it avoided potential errors in vocabulary reclassification.

Following these procedures, we adapted and expanded the existing BNC/COCA word lists by adding related members to the word families in the 25 base word lists and the four supplementary lists. Subsequently, Range was employed to reanalyze the expanded BNC/COCA word lists, leaving 3,780 word types not included in the lists. The remaining vocabulary included a large number of loan words, particularly Latin loan words (e.g., *Abies*), English vocabulary closely related to forestry (e.g., *abaca*), and a small number of general English words (e.g., *abutting*). The remaining words were highly specialized, characterized by 2,149 forestry-specific Latin loan words. Thus, this study classified the remaining vocabulary into four additional word lists: the loan word list (excluding Latin loan words) (basewrd35), forestry Latin loan word list (basewrd36), forestry English word list (basewrd37), and general English word list (basewrd38). The basewrd36 and basewrd37 consist of highly specialized words, which are fully technical vocabulary, used exclusively within the forestry domain, such as *Quercus* and *sylvestris*. The basewrd36 includes Latin scientific genus names of plants, animals, and fungi, while the basewrd37 comprises specialized forestry words in English. They constitute the specialized vocabulary found in the FAC but are not in the BNC/COCA word lists. These words play a crucial role in the lexicalization of discipline-specific knowledge and may pose challenges for ESP learners in acquiring and mastering specialized vocabulary [8].

## 4. Results and discussion

### 4.1 Lexical distribution of the forestry academic corpus

The first research question focuses on the lexical coverage and frequency distribution of the expanded BNC/COCA word lists in the FAC as a whole, as shown in Table 3.

**Table 3:** Lexical coverage of FAC across the BNC/COCA word lists.

Basewrd	Tokens	Tokens%	Cumulative coverage	Types	Types%
1	2,057,736	59.03	59.03	4,260	6.13
2	472,159	13.55	72.58	4,071	5.86
3	347,360	9.97	82.55	**4,101**	**5.9**
4	105,962	3.04	85.59	2,563	3.69
5	52,963	1.52	87.11	1,945	2.8
6	33,264	0.95	88.06	1,549	2.23
7	20,762	0.60	88.66	1,171	1.68
8	20,052	0.57	89.23	991	1.42
9	10,548	0.30	89.53	766	1.1
10	6,783	0.19	89.72	617	0.89
11	7,624	0.22	89.94	594	0.85
12	4,973	0.14	90.08	450	0.65
13	3,744	0.11	90.19	377	0.54
14	4,930	0.14	90.33	376	0.54
15	4,314	0.12	90.45	389	0.56
16	3,040	0.09	90.54	318	0.46
17	**8,568**	**0.25**	90.79	271	0.39
18	1,824	0.05	90.84	251	0.36
19	2,905	0.08	90.92	211	0.3
20	1,778	0.05	90.97	201	0.29
21	2,282	0.07	91.04	168	0.24
22	1,858	0.05	91.09	133	0.19
23	1,261	0.04	91.13	146	0.21
24	543	0.02	91.15	114	0.16
25	672	0.02	91.17	95	0.14
26–30	0	0.00	91.17	0	0
31	131,864	3.78	94.95	23,606	33.96
32	30,257	0.87	95.82	526	0.76
33	66,965	1.92	97.74	12,416	17.86
34	55,590	1.59	99.33	3,058	4.4
35	291	0.01	99.34	104	0.15
36	12,875	0.37	99.71	2,149	3.09
37	9,272	0.27	99.98	1,403	2.02
38	667	0.02	100	124	0.18
**Total**	**3,485,686**	**100**	**100**	**69,514**	**100**

As can be seen from Table 3, vocabulary in the FAC is widely distributed across all the 25 base word lists and 4 supplementary word lists in the expanded BNC/COCA word lists. The expanded BNC/COCA word lists collectively account for 99.33% of the total coverage. The first three base word lists account for 59.03%, 13.55%, and 9.97% of the total tokens in the corpus, respectively. From the fourth base word list, the coverage sharply drops to 3.04% with subsequent word lists contributing progressively smaller percentages. From the eighth base word list, with every additional 1,000 word families, the coverage increases by less than 0.5%. Originally, the 25 base word lists in the BNC/COCA word lists were ranked in descending order based on their frequency in the general English corpus. However, the expanded BNC/COCA word lists show some differences in the coverage of tokens and types in the FAC.

On the one hand, the percentages of the tokens of base word lists do not always decrease linearly but with some exceptions. Notably, basewrd17, with a coverage of 0.25%, even exceeds basewrd10 (0.19%). Further investigation reveals that some words in the basewrd17 are frequently used in the FAC, and they are names of two sub-disciplines in forestry, namely *agroforestry* and *dendrochronology*. These two technical words, which are “characterized by the absence of exact synonyms, resistance to semantic change, and a very narrow range” [46], and are “recognizably specific to a particular field” [47], show that technical words also appear in the 25 base word lists. This finding aligns with studies by Liu and Lei [48] and Hsu [49], which reported that technical vocabulary also includes words from general vocabulary and general academic vocabulary.

On the other hand, the four supplementary word lists in the BNC/COCA word lists have different percentages of coverage in the corpus. The proper noun list (basewrd31) has the highest percentage of the tokens at 3.78%, exceeding the coverage of basewrd4. The list of proper nouns mainly includes personal names (e.g., *Aakala*, *Akayla*, *Wang*), place names (e.g., *China*, *Japan*, *California*), and river names (e.g., *Chesuncook*). The transparent compounds list (basewrd33) and the acronyms list (basewrd34) account for 1.92% and 1.59% of tokens, respectively, both higher than basewrd5. The marginal words list (basewrd32) has the lowest percentage at 0.87% in forestry academic texts. The coverage percentage of proper nouns in the FAC is much higher than the 2.31%in business textbooks [7], 2.37% in business research articles [7], 1.17% in engineering textbooks [10], and 2.67% in TCM [13]. This indicates that the FAC texts use proper nouns extensively.

Table 3 also displays the coverage of four new word lists developed from the corpus. Notably, the loan word list (basewrd35) and the general word list (basewrd38) exhibit relatively low coverage rates of 0.01% and 0.02% respectively. However, two forestry-specific word lists, namely the forestry Latin loan word list (basewrd36) and the forestry English word list (basewrd37), constitute 0.37% and 0.27% of the overall running text, respectively. The coverage of the Latin loan word list surpasses that of the basewrd9 in the expanded BNC/COCA base word lists, and the coverage of the forestry English word list is nearly equivalent to the basewrd9. These figures demonstrate that discipline-specific vocabulary [50], or technical vocabulary [48], makes up a substantial proportion of texts in academic disciplines and technical fields [51–53]. Furthermore, the two forestry-specific word lists account for 3.09% and 2.02% of the total word types in the corpus, highlighting the notable proportion of unique vocabulary. This observation suggests a significant lexical richness associated with forestry-specific Latin loan words and English words within the corpus [54].

### 4.2 Lexical demands of the forestry academic texts

The lexical demands identified in lexical profile studies are typically described as the top *n* word families plus the four supplementary word lists from BNC/COCA word lists [35]. It has become standard practice to assume that the four supplementary word lists are unproblematic for readers [55]. Specifically, when recommending a vocabulary learning target, it is often assumed that if readers have mastered at least the most frequent 3,000 word families, they will also be able to comprehend the words from the four supplementary word lists in the BNC/COCA word lists, and these can be treated as known items. The second research question addresses not only lexical demands of forestry academic texts as a whole. Specifically, it examines the vocabulary required to achieve 95% and 98% coverage of the FAC. In addition, it calculates the lexical demands of the forestry research articles and textbooks separately, as well as those of the eight sub-disciplines within forestry.

As shown in Table 4, the top 3,000 word families in the expanded BNC/COCA word lists, along with four supplementary word lists including proper nouns, marginal words, acronyms, and transparent compound words provide 90.70% coverage. To achieve 95% coverage, learners need to master the top 5,000 word families along with the four supplementary word lists. To attain 98% coverage, learners have two options: mastering the top 11,000 word families along with the four supplementary word lists, or mastering the top 8,000 word families along with the four supplementary word lists and two forestry-specific word lists. Comparatively speaking, the FAC appears to be more lexically demanding than those in business [7] and engineering [10], and less demanding than physics [18], marine engineering [9], and TCM [13], as shown in Table 1.

**Table 4:** Cumulative coverage for the forestry texts.

Basewrd	Cumulative coverage%	Basewrd	Cumulative coverage%
31–34	8.16	31–34	8.16
1	67.19	36–37	8.80
2	80.74	1	67.83
3	90.71	2	81.38
4	93.75	3	91.35
**5**	**95.27**	4	94.39
6	96.22	**5**	**95.91**
7	96.82	6	96.86
8	97.40	7	97.46
9	97.70	**8**	**98.04**
10	97.89	9	98.34
**11**	**98.11**	10	98.53

Although the gap between 95% and 98% coverage is only 3%, the difference in lexical demands between the two thresholds is more significant than it may appear [28]. In the FAC, the lexical demands increase from 5,000 to 11,000 word families, adding 6,000 word families. This substantial increase is due to the fact that beginning from basewrd6, the coverage of each additional BNC/COCA base word list is relatively low, contributing less than 1% for every additional 1,000 word families. However, the two forestry-specific word lists, basewrd36 and 37, comprising 2,149 and 1,403 word types respectively, contribute 0.64% coverage. The vocabulary in these two forestry-specific word lists includes fewer word types compared to the base word lists, thereby offering greater efficiency. In other words, to reach 98% coverage, it is more effective for learners to focus on mastering the two forestry-specific word lists, which reduces the need to master the next three base word lists (basewrd9, 10, and 11), totaling 3,000 word families (an overwhelming 9,231 word types). The two forestry-specific word lists provide more targeted vocabulary, enabling ESP learners to focus on the most important words in their specialized discipline. This targeted approach serves as a shortcut reducing the amount of overall lexical demand, which is particularly beneficial given that academic disciplines usually have heavy lexical demands [1,34].

Table 5 presents the lexical demands of the forestry research articles and textbooks. As shown in the table, achieving 95% coverage in both cases requires the mastery of the top 5,000 word families along with four supplementary word lists in the expanded BNC/COCA. At the 98% coverage level, research articles are more lexically demanding than textbooks. To achieve 98% coverage for textbooks, learners would need to master 10,000 word families and the four supplementary word lists. However, for research articles, the requirement increases significantly to 12,000 word families plus the four supplementary word lists. This finding is consistent with those from earlier studies [7,9,10,18] as shown in Table 1, which demonstrates that research articles have higher lexical demands than textbooks.

**Table 5:** Cumulative coverage for the forestry research articles and textbooks.

Basewrd	Research articles	Textbooks	Basewrd	Research articles	textbooks
31–34	10.31	5.97	31–34	10.31	5.97
1	66.39	68.03	36–37	11.05	6.50
2	79.77	81.75	1	67.11	68.58
3	90.43	91.01	2	80.49	82.3
4	93.72	93.79	3	91.15	91.56
5	**95.19**	**95.36**	4	94.44	94.34
6	96.07	96.39	5	**95.91**	**95.91**
7	96.65	97.00	6	96.79	96.94
8	97.25	97.55	7	97.37	97.55
9	97.50	97.9	8	97.97	**98.1**
10	97.69	**98.1**	9	**98.22**	98.45
11	97.90	98.32	10	98.41	98.65
12	**98.07**	98.44	11	98.62	98.87

Given the high occurrence of specialized Latin loan words and forestry-specific English vocabulary in the corpus, we recommend that learners study the two forestry-specific word lists, along with the expanded BNC/COCA word lists. This approach could reduce the lexical demands to the top 8,000 word families and four supplementary word lists for forestry textbooks, as well as the top 9,000 word families plus four supplementary word lists for research articles.

**Fig 1:**
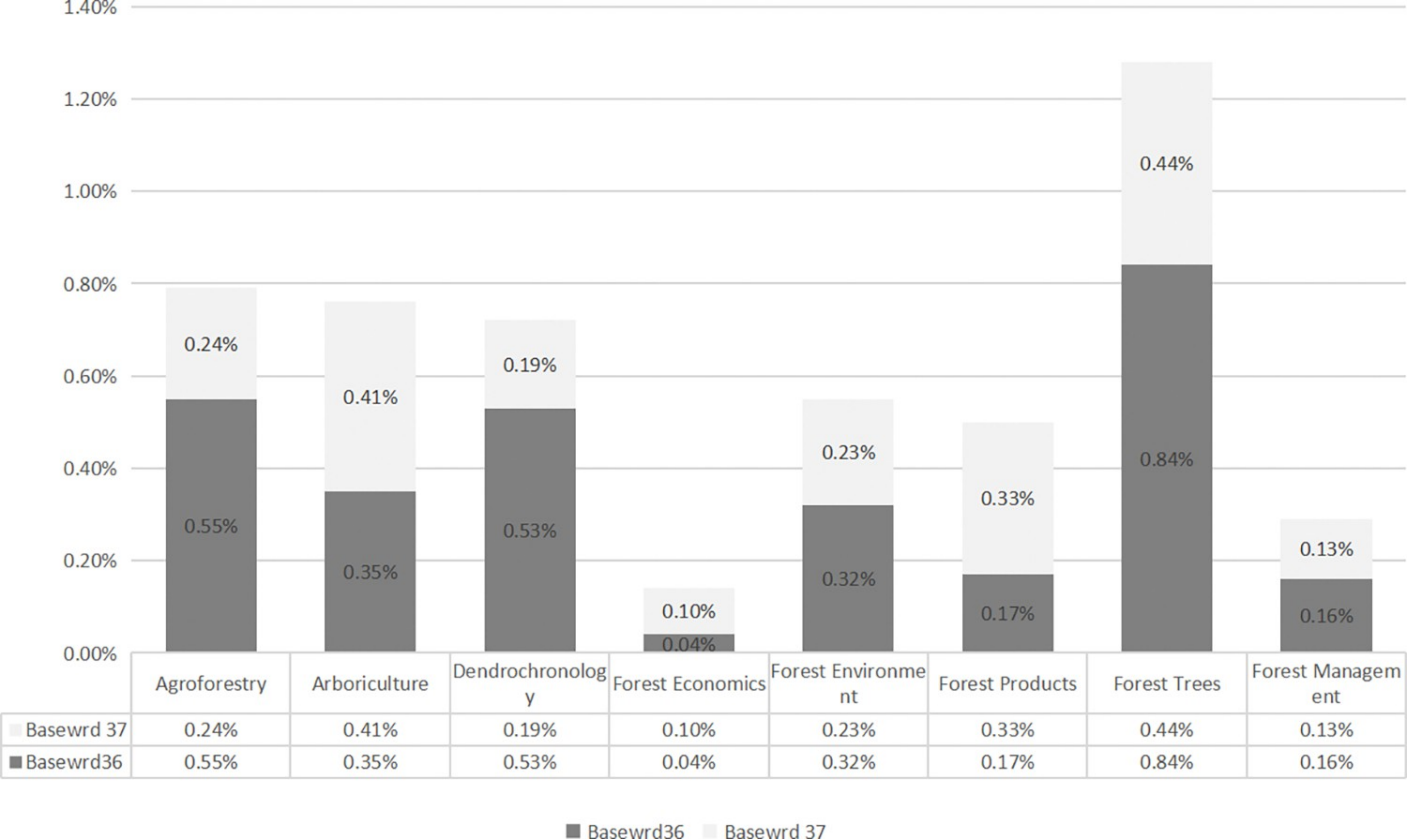
Coverage of two forestry-specific word lists in sub-disciplines.

Furthermore, this study explored the lexical demands across eight sub-disciplines of forestry to analyze whether there are differences among them. Given the observed specialization and significance of the forestry Latin loan word list and the forestry English word list, the analysis initially focuses on the coverage of these two highly specialized word lists within different sub-disciplines of forestry. The proportions of the basewrd36 and basewrd37 across eight sub-disciplines are illustrated in Fig 1.

As shown in Fig 1, the proportion of Latin loan words in forestry is the highest in forest trees (0.84%), followed by agroforestry (0.55%) and dendrochronology (0.53%), with the lowest proportion found in forest economics (0.04%). This is understandable as forest trees contains a large number of tree species, such as *acer*, *Pinus*, and *Salix*. In contrast, forest economics focuses on the laws and applications of economic relationships and activities related to the production, exchange, distribution, and consumption of material resources in forestry [56], thus resulting in a relatively lower proportion of Latin loan words. The nature of forest economics also explains the low proportion of forestry English words in this sub-discipline (0.10%). Conversely, the highest proportion of forestry English words is found in forest trees (0.44%), followed closely by arboriculture (0.41%). When the proportions of the forestry Latin loan word list and the forestry English word list are combined, the highest percentage is observed in forest trees, followed by agroforestry, while the lowest percentage is observed in forest economics.

Table 6 presents the lexical demands for achieving 95% and 98% coverage in each sub-discipline. Coverage 1 represents the lexical demands of eight sub-disciplines in the order of the expanded BNC/COCA word lists. Only the four supplementary word lists were placed at the forefront, while the two forestry-specific word lists developed in this study were calculated last. Meanwhile, Coverage 2, in contrast, prioritizes the two forestry-specific word lists, which are followed by the four supplementary word lists and the 25 base word lists from the expanded BNC/COCA word lists. That is to say, coverage 2 emphasizes the importance of the forestry Latin loan word list and the forestry English word list over the 25 base word lists in the calculation of lexical demands. From coverage 1 in Table 6, it is clear that learners who master the top 6,000 word families along with the four supplementary word lists can achieve 95% coverage in 7 out of the 8 sub-disciplines. The lowest lexical demands are found in forest economics, requiring 4,000 word families, while forest trees exhibits the highest demands at 8,000 word families. To achieve 98% coverage, the lowest lexical demands remain in forest economics at 6,000 word families, while both forest trees and agroforestry require a substantial 17,000 word families individually. These findings highlight the significant differences in lexical demands across the eight sub-disciplines of forestry.

**Table 6:** Cumulative coverage for the forestry sub-disciplines.

Sub-discipline	95% coverage1	95% coverage2	98% coverage1	98% coverage2
Agroforestry	6,000	5,000	17,000	11,000
Arboriculture	5,000	5,000	11,000	8,000
Dendrochronology	5,000	5,000	11,000	8,000
Forest Economics	4,000	4,000	6,000	6,000
Forest Environment	5,000	5,000	10,000	8,000
Forest Products	6,000	5,000	11,000	9,000
Forest Trees	8,000	6,000	17,000	11,000
Forest Management	5,000	5,000	8,000	8,000

When comparing coverage 1 and coverage 2, the disparity between the two is more pronounced at the 98% coverage level. To achieve 98% coverage in each sub-discipline, it is more effective for learners to follow coverage 2, which involves mastering the two forestry-specific word lists. This approach reduces the lexical demands by 2,000 to 6,000 word families across the sub-disciplines. For instance, the lexical demands of agroforestry and forest trees, which are the highest at 17,000 word families as shown in coverage 1, decrease to 11,000 in coverage 2, with 6,000 word families reduced. In other words, mastering the two forestry-specific word lists would reduce the lexical demands by 6,000 word families to reach the 98% threshold. This finding underscores the importance of the forestry Latin loan word list and the forestry English word list in sub-discipline with high lexical demands.

### 4.3 Comparison of forestry vocabulary with general and general academic vocabulary

Based on the preceding discussion, this study finds that forestry academic texts are lexically demanding, requiring learners to acquire a substantial vocabulary size, especially at the 98% coverage level. Forestry academic vocabulary not only comprises a large number of words that could be added to the existing BNC/COCA word lists, but also includes words that are unrelated to any word families in the BNC/COCA word lists, notably manifested in the forestry-specific Latin loan word list and the English word list. These two word lists exhibit high coverage rates within the forestry academic texts, particularly in the sub-disciplines of forest trees and agroforestry, (0.84% and 0.55%, respectively). That is to say, the vocabulary in forestry academic texts is quite different from that of general English. Therefore, it is needed to explore the distinctions between forestry vocabulary, general vocabulary, and general academic vocabulary. This exploration addresses the critical question: “Which words should be learned, and in what order should vocabulary be acquired?”. This question arises after determining the lexical demands for comprehension of forestry academic texts, as indicated by the 95% and 98% lexical coverage thresholds.

To compare the differences between forestry academic vocabulary and general English, we applied the framework proposed by Schmitt and Schmitt [57]. In their framework, the first three 1,000 word families (basewrd1-3) from the BNC/COCA word lists are categorized as high-frequency vocabulary. This categorization contrasts with the traditional view, which considers the most frequent 2,000 word families as general high-frequency vocabulary, a perspective influenced largely by the size of West’s GSL [47,58]. Additionally, word families from 4,000 to 8,000 (basewrd4-8) are classified as mid-frequency vocabulary, and those beyond 9,000 (basewrd9-25) are categorized as low-frequency vocabulary, as illustrated in the second column of Table 7. Based on their coverage, the word lists from the FAC corresponding to each frequency band are provided in the third column of Table 7.

**Table 7:** Comparison of frequency bands in BNC/COCA word lists and FAC.

Frequency bands	BNC/COCA word lists	FAC
High-frequency vocabulary	basewrd1,2,3	basewrd1, 2, 3
Mid-frequency vocabulary	basewrd4,5,6,7,8	basewrd4,5,6,7,8
Low-frequency vocabulary	basewrd9, 10, 11, 12, 13, 14, 15, 16, 17, 18, 19, 20, 21, 22, 23, 24, 25	basewrd36, 9, 37, 17, 11, 10, 12, 14, 15, 13, 16, 19, 21, 18, 20, 22, 23, 24, 25

The results of this study support Schmitt and Schmitt’s findings [57], as high-frequency vocabulary performed effectively in forestry academic texts. As discussed in Table 3, the first three 1,000 word families of the expanded BNC/COCA word lists contributed 82.55% to the coverage of FAC. The coverage of the word lists in the mid-frequency (basewrd4-8) drops dramatically to 6.68%. It is worth noting that the forestry Latin loan word list (basewrd36) and the forestry English word list (basewrd37) are positioned at the forefront of the low-frequency band. That is to say, in comparison to the frequency bands of BNC/COCA word lists of general English, the order of base word lists within the low-frequency band in the forestry academic corpus is different. Moreover, this study highlights the relatively small sizes of the forestry-specific lists compared to basewrd9 (3,233 word families), basewrd8 (3,469 word families) and basewrd7 (3,748 word families). This raises an important question regarding the categorization of mid-frequency vocabulary, specifically whether the 4,000 to 8,000 word families in FAC should be classified as mid-frequency vocabulary. This issue is particularly significant because of the extensive coverage provided by the two forestry-specific word lists. This finding supports the idea that discipline-specific word lists are better aligned with the lexical demands of ESP learners [59–61], and underscores the heavy lexical demands of the ESP texts.

Another notable observation is related to basewrd17. As discussed in the previous section, most of the coverage of basewrd17 is attributed to the two technical words *agroforestry* and *dendrochronology*. That is to say, only a small number of the items in the low-frequency bands are truly useful to the forestry learners. These two technical words greatly influence the lexical demands of the sub-discipline of agroforestry and forest trees. Thus, in a linear sequence, basewrd17 is needed to achieve 98% coverage of these two sub-disciplines, as it provides a relatively higher coverage rate within the low-frequency word lists.

Given the significance of the forestry-specific word lists, this study casts doubt on the overall effectiveness of the BNC/COCA word lists in their existing sequence for ESP learners with highly specialized language needs. The results also highlight the remarkable utility of the forestry Latin loan word list (basewrd36) and the forestry English word list (basewrd37), and lend support to the suggestion for a more focused discipline-specific word list, or technical word list for learners of forestry academic English. This kind of word list allows for the inclusion of terms like *agroforestry* and *dendrochronology*. The specialized word list, or technical word list, tailored to the needs of ESP learners could provide a more effective learning return for them [59,60].

**Fig 2:**
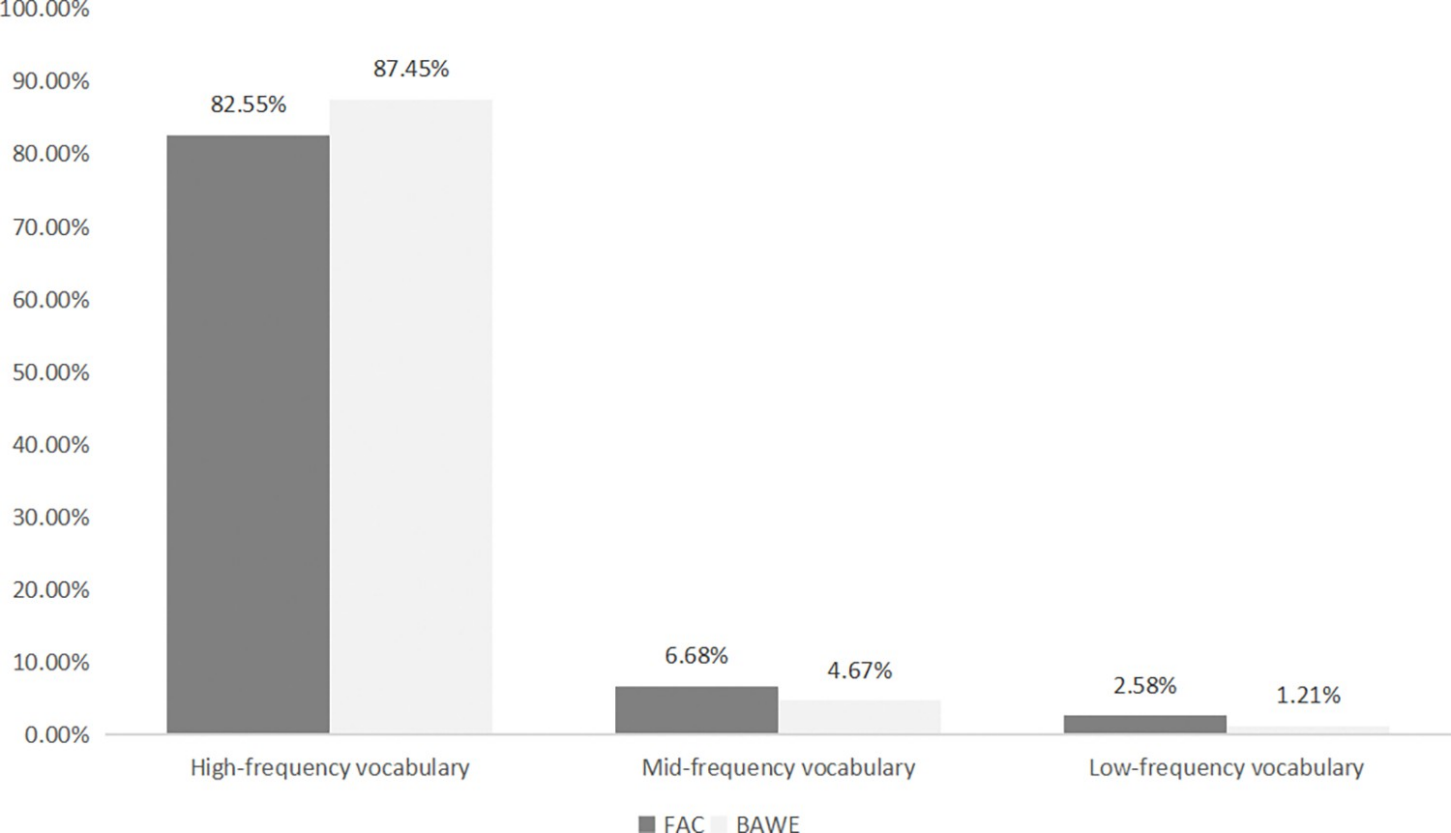
Comparison of high-, mid- and low-frequency vocabulary in FAC and BAWE.

Fig 2 presents the coverage of high-frequency, mid-frequency, and low-frequency bands in both the FAC and the BAWE corpus. While the FAC focuses on academic language used by forestry experts, the BAWE corpus includes a broader range of student academic writing across various fields, offering valuable insights into general academic writing practices [45]. The comparison of vocabulary frequency distributions between these two corpora highlights the distinctive features of forestry-specific language, while also illustrating the broader academic vocabulary shared across disciplines.

Fig 2 reveals that the coverage of high-frequency vocabulary (basewrd1-3) in the FAC (82.55%) is lower than that in the BAWE corpus (87.47%). In contrast, the coverage of mid-frequency vocabulary (basewrd4-8) and low-frequency vocabulary (basewrd9-25) in FAC (6.68% and 2.58%, respectively) is higher than that in the BAWE corpus (4.67% and 1.21%, respectively). This indicates that forestry academic texts contain a lower proportion of high-frequency vocabulary, and a higher proportion of mid- and low-frequency vocabulary compared to general academic English texts, implying greater lexical difficulty in forestry academic English. Moreover, in the low-frequency band, the two forestry-specific word lists account for 0.03% in the BAWE corpus and 0.64% in the FAC, which further underscores the difficulty and specialization of forestry academic texts, and highlights their specialized nature.

## 5. Conclusion

Using Range and the BNC/COCA word lists, this study focuses on the frequency distributions, lexical demands, difficulty and diversity of forestry academic texts as a specialized discipline. The findings are as follows: (1) Forestry academic vocabulary exhibits a notable degree of complexity and richness. While the existing BNC/COCA word lists constitute a significant portion of the FAC, many additional words have been added to the existing 1–25 base word families and four supplementary word lists of the BNC/COCA word lists. Furthermore, two forestry-specific word lists, the forestry Latin loan word list and the forestry English word list, were also developed. (2) A vocabulary of at least top 5,000 word families and four supplementary word lists from the expanded BNC/COCA word lists is needed to achieve 95% coverage. To achieve 98% coverage, the requirement increases to 11,000 word families and four supplementary word lists. However, this can be reduced to 8,000 word families if the two forestry-specific word lists are prioritized. (3) Lexical demands are diversified across different genres within the FAC. Research articles are more lexically demanding than forestry textbooks, particularly at the 98% coverage level. Significant differences in lexical demands were also observed among the eight sub-disciplines of forestry. While forestry economics exhibits the lowest lexical demands, forest trees has the highest, followed closely by agroforestry. The two forestry-specific word lists are especially crucial for the sub-discipline of forest trees and agroforestry, as they can reduce the lexical demands for 98% coverage from 17,000 to 11,000 word families. (4) Forestry academic vocabulary, distinct from general vocabulary and general academic vocabulary, contains a higher proportion of mid- to low- frequency words, with an emphasis on the forestry Latin loan word list, the forestry English word list, and the basewrd17 in low-frequency band.

This study may serve as a guide for vocabulary learning and teaching in forestry, particularly in setting appropriately sized vocabulary goals and establishing sequence for vocabulary learning during a particular phase of specialized English learning. First, the results of frequency distribution indicate that the most frequent 3,000 word families are crucial for vocabulary learning in forestry academic English. Secondly, this study also emphasized the importance of prioritizing forestry-specific words. This includes the two forestry- specific word lists outside the BNC/COCA lists, as well as technical words in the 1–25 word families in the BNC/COCA word lists. Thirdly, learners in forestry academic English face a relatively large vocabulary burden, requiring a vocabulary size of at least 5,000 word families along with four supplementary lists. Fourthly, vocabulary learning in forestry should take genre variation into account to have a gradual difficulty in proceed through forestry textbooks, journal articles and the eight sub-disciplines, as they show differences in lexical demands.

There are several limitations to this study. One limitation is that the corpus comprises only the written texts, including spoken input would provide additional insights into the profile of the vocabulary in the FAC. A second limitation is that the focus on single word units, as understanding individual words does not necessarily ensure comprehension of phrase as a whole.

Further research is encouraged to develop and evaluate a more comprehensive forestry-specific academic word list, or forestry technical word list, encompassing high-, mid-, and low-frequency terms. This would provide learners and teachers a possible shortcut to vocabulary learning in this discipline. Such research in the future should also consider the multiword units within the FAC to explore vocabulary beyond the single-word units.

## Supporting information

S1 Raw data The corpus utilized in this study was compiled from text data obtained through the CABI (Centre for Agriculture and Biosciences International) database (https://www.cabi.org), accessed via an institutional subscription provided by Bangor College, Central South University of Forestry and Technology. (ZIP)
